# The global effect of maternal education on complete childhood vaccination: a systematic review and meta-analysis

**DOI:** 10.1186/s12879-017-2890-y

**Published:** 2017-12-28

**Authors:** Jennifer Forshaw, Sarah M. Gerver, Moneet Gill, Emily Cooper, Logan Manikam, Helen Ward

**Affiliations:** 1 School of Public Health, Imperial College London, South Kensington Campus, London, SW7 2AZ UK; 2 St George’s, University of London, Cranmer Terrace, London, SW17 0RE UK; 3 UCL Great Ormond Street Institute of Child Health, 30 Guildford Street, London, WC1N 1EH UK

**Keywords:** Maternal education, Child health, Vaccination, Immunisation

## Abstract

**Background:**

There is an established correlation between maternal education and reduction in childhood mortality. One proposed link is that an increase in maternal education will lead to an increase in health care access and vaccine uptake. Vaccinations are a central preventative child health tool, therefore demonstrating the importance of understanding factors that can improve coverage. This review aims to establish if there is a correlation between increasing maternal education and vaccine uptake and if this varies between continents, setting and time.

**Methods:**

An electronic database search was conducted using Medline Ovid, Embase and The Cochrane Library using a combination of keywords and appropriate MeSH terms for maternal education and child vaccination. Bibliographies were also hand searched. Data was extracted and entered onto a Microsoft Excel spreadsheet and analysed using STATA 13.0 software. The primary outcome of effect size of maternal education on completion of childhood vaccinations was analysed at different levels. Secondary outcomes were explored using subgroup analyses of differences between continents, rural or urban settings, and dates.

**Results:**

The online search yielded 3430 papers, 37 were included in this study. The analysis showed increasing child vaccination uptake with increasing maternal education. Overall, analysis showed that the odds of full childhood vaccination were 2.3 times greater in children whose mother received secondary or higher education when compared to children whose mother had no education. There was large variability in the effect size between the studies included.

**Conclusions:**

Improving maternal education is important for increasing childhood vaccination uptake and coverage. Further research is needed in higher income countries.

**Trial registration:**

PROSPERO Registration No: CRD42016042409.

## Background

Despite the fact more children than ever are being vaccinated, millions of children each year fail to receive the complete routine immunization schedule [1]. Although the reason for this is likely multifactorial, it has been demonstrated that there is an association between maternal education and vaccination uptake [2, 3].

Childhood vaccinations are imperative for decreasing childhood mortality [1]. For this reason, global initiatives such as the Expanded Program on Immunization (EPI) and the Global Alliance for Vaccine and Immunization (GAVI) have been put in place, outlining essential vaccinations and reinforcing their uptake [4–6]. Despite this, it is estimated that 1.5 million children under 5 years die from vaccine-preventable diseases annually [7]. Although literature has shown low caregiver education to be a common variable for under or non-immunization of children, there is no research to confirm whether it is a consistent finding and the overall effect size has not been established [2, 3, 8].

The main aim of this study was to establish the global effect of maternal education on childhood vaccination in those under 12 years by quantifying the association between increasing maternal education and vaccine coverage in children, and assessing the variation in effect of maternal education by continent, setting, and over time.

## Methods

### Protocol, eligibility criteria, information sources and search

Medline, Embase, and the Cochrane Library were electronically searched on the 29th June 2016 using a combination of keywords and MeSH terms describing maternal education and child vaccination uptake. The search was restricted to English language and limited to those published between 1990 and 2016.

### Study selection, data collection and data items

Observational studies of mothers with children under 12 years were included. Studies had an exposure variable of maternal education which is cross comparable such as “level of schooling achieved” or “literate versus illiterate” with a comparison group within the article.

The primary outcome assessed was completion of the full national or EPI schedule. Secondary outcomes were difference between continents, settings and dates.

Studies were subject to the following exclusion criteria: vaccine uptake not presented as raw, unadjusted data; unable to access the full text; review or narrative design; random control trials; case control trials not proportionate to the total population; studies where the exposure was another variable but maternal education was adjusted for in the analysis; studies with the outcome of specific vaccines, receipt of any vaccine, or vaccines not in the EPI.

Two authors (JF and MG, or EC and MG) independently screened all the titles. Abstracts were reviewed of potentially relevant articles, and full texts were retrieved to ascertain whether the inclusion criteria were fully met. Discrepancies were discussed until a consensus was reached. Data was extracted from included papers regarding study characteristics, including publication information (author and year), study country, setting, design, period, population total, children’s age, maternal education parameter and vaccine types. The number of children per maternal education level, the number of children fully vaccinated per maternal education level, and the percentage of children fully vaccinated per maternal education level were extracted for data analysis.

When the paper presented more than one set of results, for example different years, locations or age-groups, the paper was split into alphabetically ordered groups. For the 2 cohort studies included, the oldest age followed in the study was used (7 months old).

### Risk of bias

Papers were assessed for quality and risk of bias using an adapted version of the certified “Quality Assessment Tool for Quantitative Studies” by the Effective Public Health Practice Project (EPHPP) [9–11]. Each study was assessed according to the representativeness of the sample, study design, controlling of confounders, blinding of exposure for cohort studies, data collection measurements, and reporting of withdrawals and drop outs for cohort studies. The articles were given a global rating of strong, moderate or weak. All studies were kept in regardless of quality due to the small number of studies available and recognition of the limitations of the scoring systems [10, 12].

### Summary measures and synthesis of results

For the meta-analysis the maternal education variables were collapsed into a binary categorical variable (“none/primary” and “secondary/higher”). In papers where there were only two categories for maternal education level and the level of education and the type of schooling received was not clear, i.e. “illiterate versus literate”, “not educated versus educated”, the educated variable was classified as “none/primary” as the level of education was not stated. For the six studies that divided papers into the categories “literate” and “illiterate” a separate meta-analysis was conducted for comparison. This is because the quality of education within countries can be highly varied, meaning we cannot conclude that a primary level education will result in maternal literacy [13]. Papers were excluded from the meta-analysis if the lowest level of education category included were “primary / secondary,” “<high school,” or “<12 years.”

A pooled odds ratio, using the collapsed categories from each included paper, was calculated using a DerSimonian-Laird [14] random effects model, as large heterogeneity was anticipated considering the differences in study characteristics, such as varied populations, healthcare, settings and education systems. The analysis was performed in Stata version 13.0 [15]**.**

Sub-group analysis was also conducted for continent, setting, and for date the study was conducted. For the setting sub-group analysis, studies which were performed at a national or regional level were removed. In the date sub-group analysis, the data set was divided into two groups based upon the year that the studies were conducted, before and after 2000 to coincide with the release of the Millennium Development Goals.

All of the extracted papers were included into the pooled estimate analysis. The maternal education levels quoted in the papers were categorised into none, primary, secondary or tertiary to get an overall percentage of children fully vaccinated for each level.

Where dichotomous variables were stated, the lowest level was taken as this was the minimum amount the woman had received. Variables of “can read and write”, “literate” and “mother educated” were categorised as primary as these skills can be achieved from primary school level. Where the paper included a variable with “less than”, the country setting was taken into consideration due to variations in levels of mandatory education between countries.

Forest plots were created for the overall analyses and for each of the stratified analyses. These showed the individual study odds ratios and 95% Confidence Intervals, the DerSimmonian-Laired pooled estimate and the I^2^-value for heterogeneity.

### Publication bias

A scatter plot of number of children included in the studies against the prevalence of fully vaccinated children was created using STATA to assess for publication bias of the included papers.

## Results

### Study selection

**Fig. 1:**
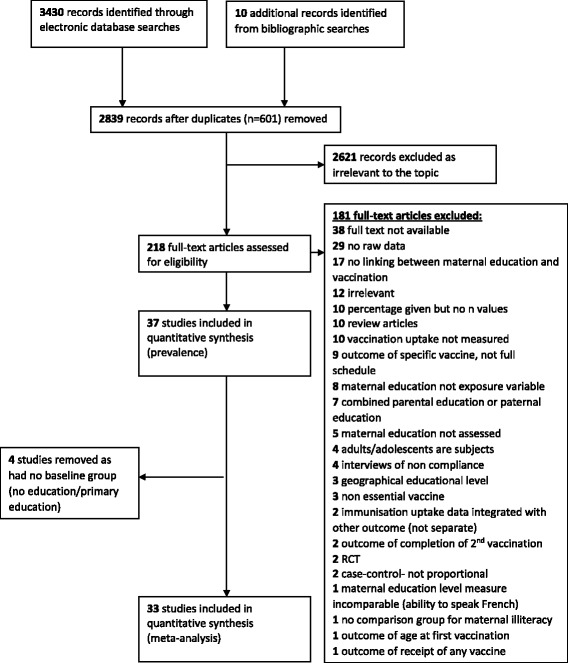
A flow diagram of study selection

The online search yielded 3430 results. Titles and abstracts were screened and duplicates or irrelevant articles were removed. In total, 218 full texts were retrieved and screened, with 37 articles being included in this review. Reasons for exclusion are outlined in Fig. 1, with the main reason being a lack of raw data.

Four papers were excluded from the meta-analysis as the lowest level of education was higher than primary.

### Study range and characteristics

Of the 37 included papers, 35 were cross-sectional studies, the remaining 2 were cohort studies. All of the data from the studies was conducted between 1989 and 2013. India had eight studies, which is the greatest total number of studies per country. When assessing by continent, 18 were undertaken in Africa, 12 in Asia, three in Europe, three in North America and one in South America. This showed a dominance of research in lower income countries. The majority of the studies were regional or national, but six studies were set in urban areas, five in rural and one study compared both. Many were population based studies, and two were conducted in a hospital setting.

Full details of the included articles are presented in Table 1 showing the characteristics of the papers included and the quality of the studies that were compared. The majority (26 studies) were of moderate quality, with only one found to be of strong quality. Ten studies scored a global score of weak but were still included in the analysis due to the small number of studies available. Most of the studies were well conducted, but their cross-sectional study design meant the global score was brought down. The sample size ranged from 220 households (with 110 children) to 21,212 children in a cross-sectional American study. The total number of children was 112,841, with a mean of 836 children and median of 190 children per study (calculated from Table 2). Of the 33 included in the meta-analysis, the total number of children was 92,192, with a mean of 2794 and a median of 693. The age range was from birth to seven years, with the majority of studies using 12–23 months as the objective population due to the EPI schedule targeting this age group [16]. The papers using demographic health survey (DHS) data were conducted on women aged 15–49 years old. On most other papers, this was not specified.

**Table 1:** Study characteristics

Reference	Country	Study setting	Study design	Study period	Population	Children’s age	Vaccine type	Maternal education parameter	Quality
Al-Sheikh et al. 1999a [17]	Iraq	Urban	Cross-sectional	1989–1994	341 families (186 urban), 662 children (326 urban)	0–2 years	BCG, DPT-OPV(3), measles, MMR, DPT-OPV(1st booster)	Illiterate; Reads and writes; Primary; Intermediate; Secondary; Institute; College; Postgraduate	Weak
Al-Sheikh et al. 1999b [17]	Iraq	Rural	Cross-sectional	1989–1994	341 families (155 rural), 662 children (336 rural)	0–2 years	Completion of BCG, DPT-OPV(3), measles, MMR, DPT-OPV(1st booster)	Illiterate; Reads and writes; Primary; Intermediate; Secondary; Institute; College; Postgraduate	
Animaw et al., 2014 [24]	Ethiopia	Region	Cross-sectional	March 2013	630 children	12–23 months	1 dose BCG, 3 doses Polio, 3 doses Pentavalant, 3 doses PCV, 1 dose Measles	None; Primary school; High school	Moderate
Antai 2009 [4]	Nigeria	National	Cross-sectional	2003	Interviews from 3725 women aged 15 to 49 years with 6029 live born children	12 months and older	BCG, Polio (3), DPT (3) and Measles vaccinations	No education; Primary; Secondary or higher	Moderate
Antai 2012 [20]	Nigeria	National	Cross-sectional	2008	24,910 women aged 15–49 years with live-born children within 5 years before the survey	12 months to 5 years	8 childhood vaccinations in the EPI – BCG, DPT 3 doses, OPV 3 doses, and measles vaccine	No education; Primary school; Secondary school or higher	Moderate
Bbaale et al. 2013 [25]	Uganda	National	Cross-sectional	2006	7591 children	12–36 months	Full vaccination, BCG, DPT, Polio, Measles vaccinations	None, primary, secondary, post-secondary	Moderate
Branco et al. 2014 [26]	Brazil	Urban	Cross-sectional	January 2010	282 children	12–59 months	1 dose BCG, 3 doses Hep B, 3 doses DTP-Hib, 3 doses OPV, 2 doses Rotavirus, 1 dose Yellow fever, 1 dose MMR	0–8 years of schooling; >8 years of schooling	Moderate
Brenner et al. 2001 [27]	USA	Urban	Cohort	August 1995 to September 1996	369 singleton births from 3 hospitals from low-income, inner-city patients	Cohort followed until 7 months	UTD at 7 months if had received 3 DTP, 3 HIB, and 2 polio vaccinations	<12 years; ≥12 years	Strong
Calhoun et al. 2014 [28]	Kenya	Region	Cross-sectional	June–July 2003	244 children	12–23 months	3 doses Polio, 1 dose BCG, 1 dose Measles, 3 doses DPT or pentavalent	Years of schooling: 0–8, 8 or more	Moderate
Chhabra et al. 2007 [29]	India	Urban	Cross-sectional	October 2003 to January 2004	693 children	24–47 months	BCG, DPT and OPV (3 primary and booster), measles and MMR	Nil; 1–8 years; >8 years	Moderate
Danis et al. 2010 [18]	Greece	National	Cross-sectional	Academic year 2004–2005	3609 parent/ guardian-child pairs 3434 pairs in the final analysis. Children in first year of Greek grammar school	6–7 years (Mean age 6.76 years)	5 doses of DTP vaccine, 5 doses of poliomyelitis vaccine, 2 doses of MMR vaccine, 3 doses of HBV vaccine and full vaccination for Hib	<9 years; 9–11 years; 12 years (high school); College/ university graduate	Moderate
Elliott et al. 2006a [30]	India	Rural	Cross-sectional	September 2003	470 families	9 months	BCG, OPV (4), DPT (3) and measles	Illiterate; Literate	Weak
Elliott et al. 2006b [30]	India	Rural	Cross-sectional	September 2003	470 families	18 months	BCG, OPV (5), DPT (4) and measles	Illiterate; Literate	
Elliott et al. 2006c [30]	India	Rural	Cross-sectional	September 2003	470 families	6 years	BCG, OPV (5), DPT (4), measles and DT	Illiterate; Literate	
Fatiregun et al. 2012 [31]	Nigeria	Region	Cross-sectional	2007	540 interviews, 525 respondents mothers of children	12–23 months	BCG, dose of measles, three doses (1,2,3) of DPT, four doses (0–3) of OPV	Primary/ secondary; Post secondary	Moderate
Fatiregun et al. 2013 [32]	Nigeria	Region	Cross-sectional	2006	1178 mothers	12–23 months	BCG, 4 doses OPV, 3 doses DPT, 3 doses Hetaptitis B	Tertiary education; Secondary education; Primary education; None	Moderate
Huq et al. 2008 [33]	Bangladesh	National	Cross-sectional	1999–2000	755 children	12–23 months	BCG and measles vaccinations and all 3 doses of the DPT and polio vaccines	Below primary; Secondary; Higher secondary	Moderate
Jahn et al. 2008 [34]	Malawi	Rural	Cross-sectional	21st August 2002 to 22nd July 2004	5418 children	Under 5 years old	BCG, OPV3, DPT3 and measles vaccine before their 1st birthday	<5 years primary; Primary 5 + years; Sec./tert.	Moderate
Kidane et al. 2003 [35]	Ethiopia	Region	Cross-sectional	2000	220 households	12–23 months	BCG, measles, 3 doses of DPT/OPV	Illiterate; Literate	Weak
Koumaré et al. 2009 [36]	Mali	Region	Cross-sectional	July 2006	750 children	12–23 months	BCG, DTCP1, DTCP2, and DTCP3 and measles	Mother not educated; Mother educated	Weak
Kumar et al. 2010 [37]	India	Hospital/ Urban	Cross-sectional	April to July 2007	325 children (148 males, 177 females) admitted to paediatrics ward at a tertiary care hospital	12–60 months	BCG, 3 doses of DPT/OPV and measles	≤primary; >primary	Weak
Luman et al. 2003 [38]	USA	National	Cross-sectional	July 2000– June 2001	21,212 children	19 to 35 months	4 doses of DPT vaccine, 3 doses of poliovirus vaccine, 1 dose of MMR vaccine, 3 or 4 doses of Hib vaccine, and 3 doses of HBV vaccine (the 4:3:1:3:3 series).	<High school; High school; >High school; College graduate	Moderate
Mohamud et al. 2014 [39]	Ethiopia	Region	Cross-sectional	10 April 2011–5 May 2011	582 households	12–23 months	1 dose BCG, 1 dose Measles, 3 doses pent/OPV before 1 year of age	Illiterate; Literate	Moderate
Odusanya et al. 2008 [40]	Nigeria	Rural	Cross-sectional	September 2006	339 mothers and children	12–23 months	BCG, 3 doses of OPV & DTP, 3 doses of HBV and measles vaccine	None/ primary; Secondary/ university	Moderate
Okoro et al. 2014 [41]	Nigeria	Region	Cross-sectional	May to December	168 children	6 months – 5 years	Full schedule (not specified)	No formal education; Primary; Secondary; Post-secondary; University	Moderate
Pati et al. 2011 [42]	USA	Urban	Cohort	June 15th 2005 to August 6th 2006	506 Medicaid-eligible mother-infant dyads	Cohort followed until 7 months	UTD at 7 months if received 3 HepB, 2 polio, at least 2 Hib, 3 PCV7 and 3 DTaP containing vaccines	Less than high school; High school; More than high school	Moderate
Phukan et al. 2008 [43]	India	Region	Cross-sectional	June and July 2003	616 children	12–23 months	6 EPI vaccines in time	Illiterate; Primary; Middle; Higher	Weak
Robert et al. 2014a [44]	Belgium	Region	Cross-sectional	2012	519 children	18–24 months	Hexavalent, pneumococcal, MMR, meningococcal C	Maximum secondary level; Higher than secondary level	Moderate
Robert et al. 2014b [44]	Belgium	Region	Cross-sectional	2012	538 children	18–24 months	Hexavalent, pneumococcal, MMR, meningococcal C	Maximum secondary level; Higher than secondary level	
Rossi et al. 2015 [45]	Zimbabwe	National	Cross-sectional	2010–2011	1031 children	12–23 months	1 dose BCG, 1 dose Measles, 3 doses of Polio, 3 doses DPT/Pentavalent	No education or primary; Secondary or higher	Moderate
Schoeps et al. 2013 [46]	Burkina Faso	Region	Cross-sectional	September 2008 – December 2009	1665 children	12–23 months	BCG, Oral Polio, Pentavalent, yellow fever, measles	Any; None	Moderate
Setse et al. 2006 [47]	Zambia	Hospital/ Urban	Cross-sectional	January 1998 and October 2000	473 children hospitalised with measles- 372 in subgroup analysis	4 and 60 months	BCG and completed the series of DTP and OPV vaccines.	Less than 7 years; 7 years; Greater than 7 years	Moderate
Sia et al. 2009 [5]	Burkina Faso	Rural	Cross-sectional	1998	805 children	12–23 months	BCG, measles, yellow fever vaccines and 3 doses of DTP and OPV	No schooling; Primary or secondary school	Moderate
Singh et al. 2000 [48]	India	National	Cross-sectional	June–October 1999	18,783 children	12–23 months	BCG, DPT, OPV, Measles	Illiterate; Primary; Middle; Higher secondary; Graduate	Weak
Singh et al. 2001 [49]	India	Region	Cross-sectional	June–October 1999	6171 children	12–32 months	BCG, DPT, OPV, Measles	Illiterate; Primary Middle; Higher secondary; Graduate	Weak
Som et al. 2010 [50]	India	Region	Cross-sectional	2002 to 2004	1279 children	12–35 months	BCG, 3 injections of DPT, 3 doses of polio (excluding polio 0) and 1 of measles	Can’t read and write; Can read and write	Moderate
Streatfield et al. 1990 [51]	Indonesia	Rural	Cross-sectional	1989	519 mother-child dyads	Under the age of 5 years	DPT, BCG, and anti-polio	Not literate; Some primary; Complete primary; Secondary school	Weak
Thang et al. 2007 [52]	Vietnam	National	Cross-sectional	2002	468 children	11–23 months	BCG vaccination 3 doses of DPT vaccine; at least 3 doses of polio vaccine; and 1 dose of measles vaccine	Illiterate; Lower primary; Completed primary; Completed secondary; Completed high school +	Moderate
Torun et al. 2006 [53]	Turkey	Region	Cross-sectional	2005	Parents of 221 children	9 month-6 years of age	<18 months completely vaccinated if had 1 dose of BCG, 3 doses of HBV, OPV and DPT and 1 dose of Measles vaccine. >18 months completely vaccinated if had booster doses for OPV and DPT vaccines	Illiterate; Graduated primary school; Graduated secondary school or higher education	Moderate
Waters et al. 2004a [54]	Cameroon	National	Cross-sectional	1998	2123 children	Younger than 3 years	By 6 weeks- 1st dose of DPT and the 2nd dose of polio vaccine; By 10 weeks- 2nd dose of DPT and the 3rd dose of polio vaccine; By 14 weeks- 3rd DPT dose; By 9 months- measles vaccine	Less than primary school; Primary school; Secondary education; Higher education	Moderate
Waters et al. 2004b [54]	Cameroon	National	Cross-sectional	2000	3582 children	Younger than 5 years		Less than primary school; Primary school Secondary education or higher education	
Yadav et al. 2004 [55]	India	Regional	Cross-sectional	June–October 1999	1481 children	12–23 months	BCG, DPT3, OPV3, Measles	Illiterate; Primary; Middle; Hr. Secondary; Graduate	Weak

Abbreviations: *UTD* up to date, *EPI* Expanded Program on Immunization, *OPV* oral polio vaccine, *BCG* bacille Calmette-Guérin (tuberculosis) vaccine**,** *DPT* diphtheria, pertussis, tetanus vaccine, *Hib haemophilus influenzae* type b, *HBV* hepatitis B virus, *MMR* measles, mumps & rubella vaccine, *DT* diphtheria and tetanus, *PCV7* pneumococcal conjugate vaccine (7-valent), *DTaP* diphtheria, tetanus and acellular pertussis vaccine, *DTCP* diphtheria, tetanus, pertussis, poliomyelitis vaccine

**Table 2:** Study results

Reference	Maternal education parameter	# children whose mothers had education level	# children who have received full vaccination schedule	% children who received full vaccination schedule (1 d.p.)	cOR for vaccination (2 d.p.)
Al-Sheikh et al. 1999a [17]	Illiterate	27	22	81.5	1
	Reads and writes	69	41	59.4	0.33
	Primary	78	42	53.8	0.27
	Intermediate	32	22	68.8	0.5
	Secondary	53	29	54.7	0.27
	Institute	43	27	62.8	0.38
	College	23	12	52.2	0.25
	Postgraduate	1	1	100	/
Al-Sheikh et al. 1999b [17]	Illiterate	143	34	23.8	1
	Reads and writes	121	34	28.1	1.25
	Primary	50	10	20	0.8
	Intermediate	5	2	40	2.14
	Secondary	7	5	71.4	8.01
	Institute	6	4	66.7	6.41
	College	4	4	100	/
	Postgraduate	0	/	/	/
Animaw et al. 2014 [24]	None	262	150	57.3	1.00
	Primary	252	211	83.8	3.84
	High school	116	100	86.2	4.66
Antai 2009 [4]	No education	2155	169	7.8	1
	Primary	805	142	17.6	2.52
	Secondary or higher	771	194	25.2	3.95
Antai 2012 [20]	No education	12,265	722	5.9	1
	Primary school	5724	1159	20.2	4.06
	Secondary school or higher	6921	2402	34.7	8.50
Bbaale et al. 2013 [25]	No education	1824	967	53.0	1.00
	Primary	4686	2484	53.0	1.00
	Secondary	896	520	58.0	1.23
	Post-secondary	185	117	63.2	1.52
Branco et al. 2014 [26]	0–8 years of schooling	151	116	76.8	1.00
	>8 years of schooling	130	117	90	2.72
Brenner et al. 2001 [27]	<12 years	145	55_a_	38	1
	≥12 years	179	77_a_	43	1.23
Calhoun et al. 2014 [28]	0–7 years of schooling	132	35	26.5	1.00
	≥8 years of schooling	23	11	47.8	2.54
Chhabra et al. 2007 [29]	Nil	378_b_	130	34.4	1
	1–8 years	106_b_	51	48.1	1.77
	>8 years	209_b_	106	50.7	1.96
Danis et al. 2010 [18]	<9 years	536	278	51.9	1
	9–11 years	429	240	55.9	1.18
	12 years (high school)	1336	859	64.3	1.67
	College/ university graduate	985	670	68	1.97
Elliott et al. 2006a [30]	Illiterate	332_b_	240	72.3	1
	Literate	139_b_	123	88.5	2.95
Elliott et al. 2006b [30]	Illiterate	318_b_	210	66	1
	Literate	127_b_	113	89	4.15
Elliott et al. 2006c [30]	Illiterate	139_b_	73	52.5	1
	Literate	49_b_	35	71.4	2.26
Fatiregun et al. 2012 [31]	Primary/ secondary	297	76	25.6	1
	Post secondary	228	94	41.2	2.04
Fatiregun et al. 2013 [32]	None	129	24	18.6	1.00
	Primary	468	128	27.4	1.65
	Secondary	523	225	43.0	3.30
	Tertiary	58	51	87.9	31.88
Huq et al. 2008 [33]	Below primary	485	307_a_	63.3	1
	Secondary	221	164_a_	74.2	1.67
	Higher secondary	49	46_a_	93.9	8.92
Jahn et al. 2008 [34]	<5 years primary	237	140	59.1	1
	Primary 5 + years	1364	903	66.2	1.36
	Sec./tert.	304	233	76.6	2.27
Kidane et al. 2003 [35]	Illiterate	92	66	71.7	1
	Literate	18	17	94.4	6.70
Koumaré et al. 2009 [36]	Mother not educated	639	376_a_	58.8	1
	Mother educated	111	73_a_	65.8	1.35
Kumar et al. 2010 [37]	≤primary	223	12	5.4	1
	>primary	92	46	50.0	17.58
Luman et al. 2003 [38]	<High school	3157	2147_a_	68.0	1
	High school	7160	5191_a_	72.5	1.24
	>High school	4375	3233_a_	73.9	1.33
	College graduate	8698	6915_a_	79.5	1.82
Mohamud et al. 2014 [39]	Illiterate	510	167	32.7	1.00
	Litterate	72	46	63.9	3.63
Odusanya et al. 2008 [40]	None/ primary	107	57	53.3	1
	Secondary/ university	232	153	65.9	1.70
Okoro et al. 2014 [41]	No formal education	12	7	58.3	1.00
	Primary	33	16	48.5	0.67
	Secondary	55	36	65.5	1.35
	Post-secondary	28	24	85.7	4.29
	University	40	32	80.0	2.86
Pati et al. 2011 [42]	Less than high school	159	63	39.6	1
	High school	119	55	46.2	1.31
	More than high school	228	101	44.3	1.21
Phukan et al. 2008 [43]	Illiterate	132	50	37.9	1
	Primary	81	41	50.6	1.68
	Middle	344	242	70.3	3.89
	Higher	59	50	84.7	9.11
Robert et al. 2014a [44]	Maximum secondary level	293	237	80.8	1.00
	Higher than secondary level	214	177	82.9	1.13
Robert et al. 2014b [44]	Maximum secondary level	296	242	81.6	1.06
	Higher than secondary level	233	197	84.4	1.29
Rossi et al. 2015 [45]	No education or primary	320	177	55.2	1.00
	Secondary or higher	711	500	70.3	1.91
Schoeps et al. 2013 [46]	None	1435	250	17.4	1.00
	Any	230	57	24.8	1.56
Setse et al. 2006 [47]	Less than 7 years	137	92_a_	67_c_	1
	7 years	114	87_a_	76_c_	1.56
	Greater than 7 years	121	105_a_	87_c_	3.30
Sia et al. 2009 [5]	No schooling	850	172_a_	20.2	1
	Primary or secondary school	48	18_a_	37.5	2.37
Singh et al. 2000 [48]	Illiterate	7337	3404_a_	46.4	1
	Primary	2946	1912_a_	64.9	2.14
	Middle	3044	2143_a_	70.4	2.75
	Higher secondary	3433	2705_a_	78.8	4.29
	Graduate	2023	1705_a_	84.3	6.20
Singh et al. 2001 [49]	Illiterate	3421	1143_a_	33.4	1
	Primary	900	496_a_	55.1	2.45
	Middle	718	442_a_	61.5	3.19
	Higher secondary	580	416_a_	71.8	5.08
	Graduate	552	442_a_	80	7.98
Som et al. 2010 [50]	Can’t read and write	400	151_a_	37.8	1
	Can read and write	879	538_a_	61.2	2.60
Streatfield et al. 1990 [51]	Not literate	78	35_a_	45.1	1
	Some primary	129	40_a_	31.1	0.55
	Complete primary	177	59_a_	33.6	0.62
	Secondary school	81	44_a_	54.9	1.48
Thang et al. 2007 [52]	Illiterate	33	13_a_	39.5	1
	Lower primary	74	37_a_	50	1.53
	Completed primary	157	100_a_	63.5	2.66
	Completed secondary	122	94_a_	77.4	5.25
	Completed high school +	83	69 _a_	82.9	7.43
Torun et al. 2006 [53]	Illiterate	31_b_	15	48.4	1
	Graduated primary school	157_b_	141	89.8	9.4
	Graduated secondary school or higher education	33_b_	31	93.9	16.53
Waters et al. 2004a [54]	Less than primary school	438	105_a_	24	1
	Primary school	603	235_a_	39	2.02
	Secondary education	473	246_a_	52	3.43
	Higher education	12	8_a_	67	6.43
Waters et al. 2004b [54]	Less than primary school	961	202_a_	21	1
	Primary school	1137	387_a_	34	1.94
	Secondary education or higher education	840	403_a_	48	3.47
Yadav et al. 2004 [55]	Illiterate	835_d_	407_a_	48.7	1
	Primary	241	180_a_	74.8	3.13
	Middle	190	142_a_	74.9	3.14
	Hr. Secondary	119	93_a_	78.2	3.78
	Graduate	96	77_a_	80.2	4.27

d.p. = decimal places

_a_ Number of children fully vaccinated calculated using available data in the paper (i.e. % uptake x total number of children)

_b_ Total number of children per maternal education level calculated from adding row total

_c_ Reverse percentage calculated from data in paper (percentage incompletely vaccinated presented)

_d_ Number of children with an illiterate mother calculated from deducting number in other levels from total population size

Maternal education levels varied between the study settings, with those set in higher income countries having higher baselines, potentially due to difference in schooling between countries. Dichotomous variables were used in 14 studies where the woman was classed as either literate or not, or above or below a set threshold.

### Data extraction

The raw results show a general increase in vaccination completion with increasing maternal education within the separate papers (Table 2). The odd ratios between the highest and lowest education levels within the studies ranged from 0.25, showing a decrease in completion, to 31.88 showing hugely increased odds of the children being fully vaccinated if the mother was more educated than the baseline group. Only two studies showed decreased odds between lowest and highest education levels, with the rest all showing a positive trend. Percentage fully vaccinated also varied widely from 1.0% to 100% with an average of 55.9% having completed the immunisation schedule. These variations are further explored by the meta-analysis.

### Meta-analysis

**Fig. 2:**
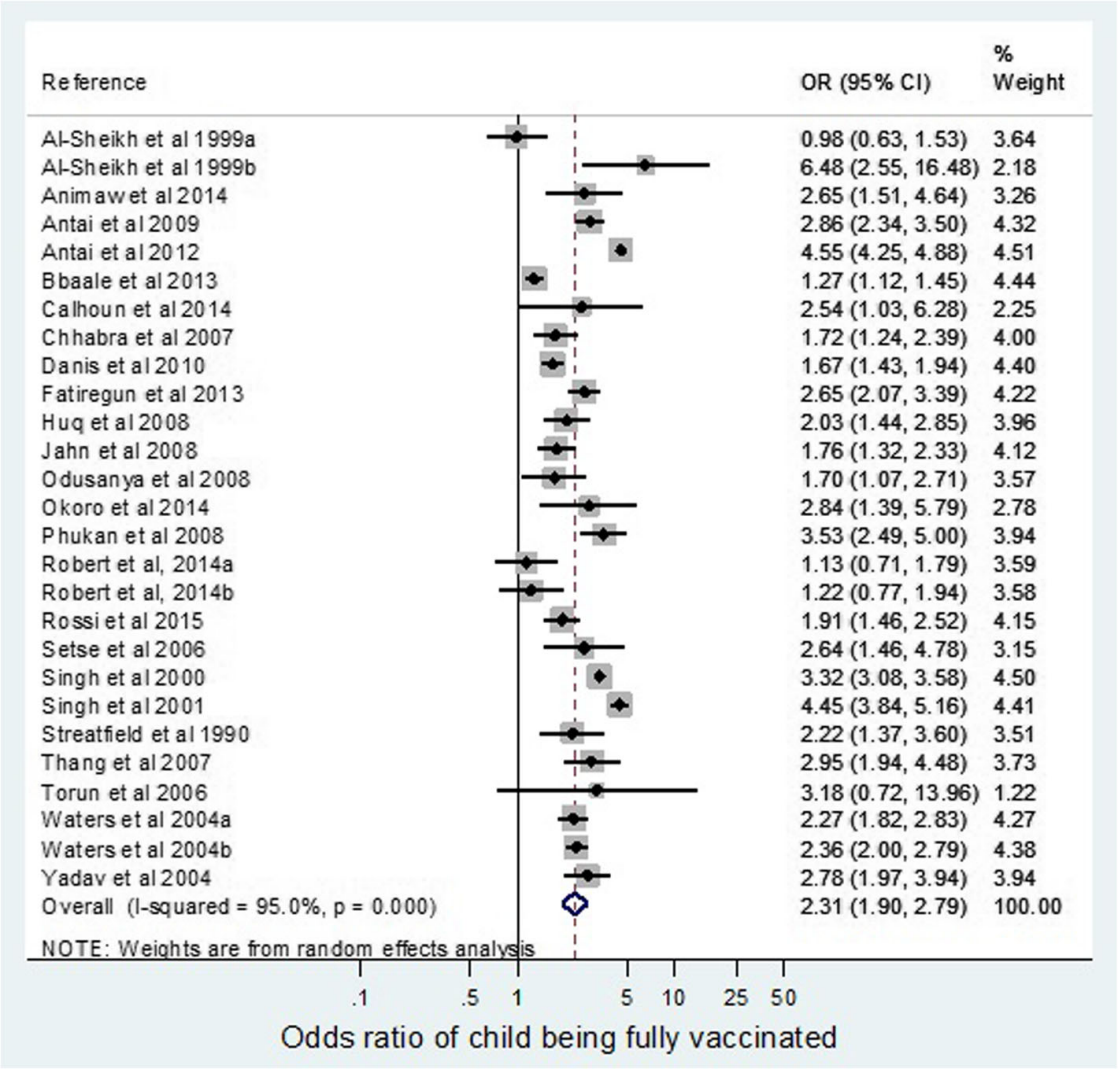
Odds ratio of children being fully vaccinated if mother educated to a secondary level compared with no or primary education

Overall, the meta-analysis showed that the odds of full childhood vaccination were 2.31 times (95% CI 1.90–2.79) greater in children whose mothers had received secondary or higher education when compared to those whose mothers had no education or primary level education (Fig. 2). Although all but four studies showed a positive effect of being highly educated, the effect size varied greatly between papers, with an overall I-squared value of 95.0% (*p* < 0.001), indicating a high level of heterogeneity.

#### Illiteracy vs. literacy

**Fig. 3:**
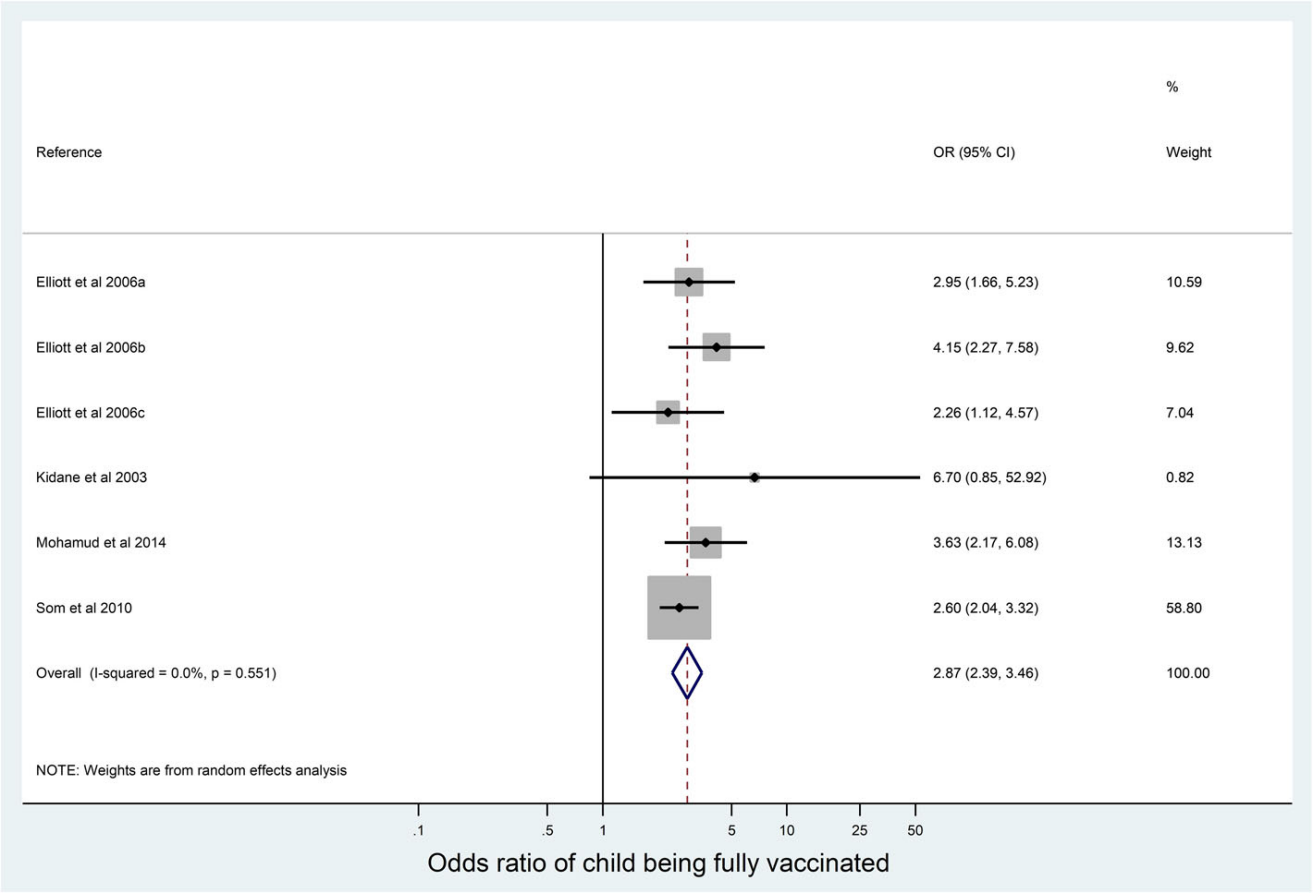
Odds ratio of children being fully vaccinated if mother is literate compared with illiterate

Figure 3 shows a separate meta-analysis of six studies which split mothers based upon whether they were literate or illiterate. It demonstrates full vaccination of children was more likely in mothers that were literate compared to illiterate, with an odds ratio of 2.87 (95% CI 2.39–3.46).

#### Continent

**Fig. 4:**
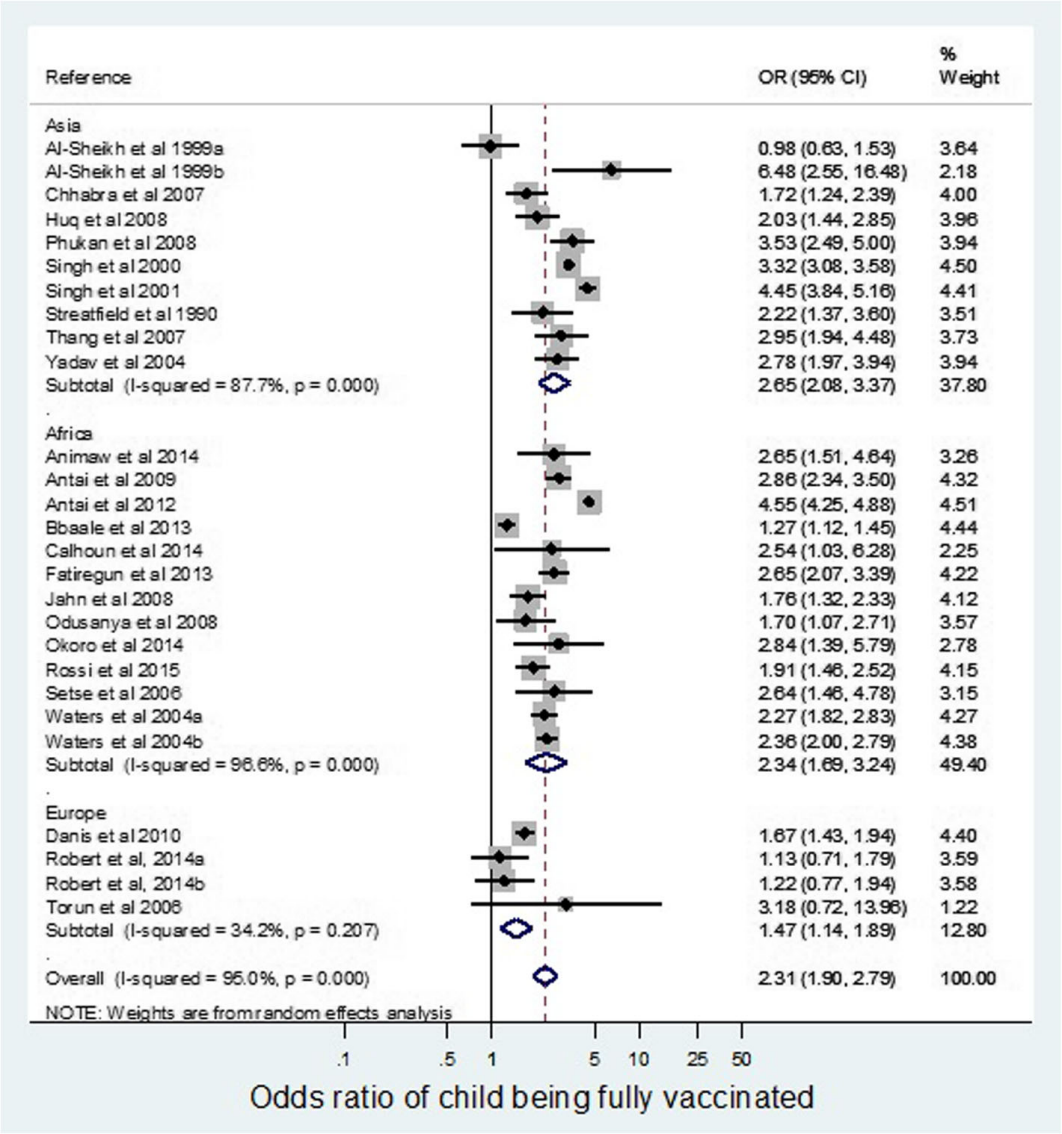
Odds ratio of children being fully vaccinated if mother educated to a secondary level compared with no or primary education, according to continent

Subgroup analysis of continents (Fig. 4) showed the overall effect size is highest in Asia, where the odds of full childhood vaccination were 2.65 times (95% CI 2.08–3.37) greater if the mother was more educated. Only one result out of 11 was not statistically significant (Al-Sheikh et al. 1999a) [17].

The overall effect for Africa was increased odds of 2.34 (95% CI 1.69–3.24) for completion of childhood vaccination with higher maternal education. There were no statistically insignificant papers in this subgroup.

The overall effect was lower in the higher income continent of Europe, with increased odds of 1.47 (95% CI 1.14–1.89) for completion of childhood vaccination with higher maternal education. Furthermore, three-quarters of European papers had statistically insignificant results, and low heterogeneity.

#### Setting

**Fig. 5:**
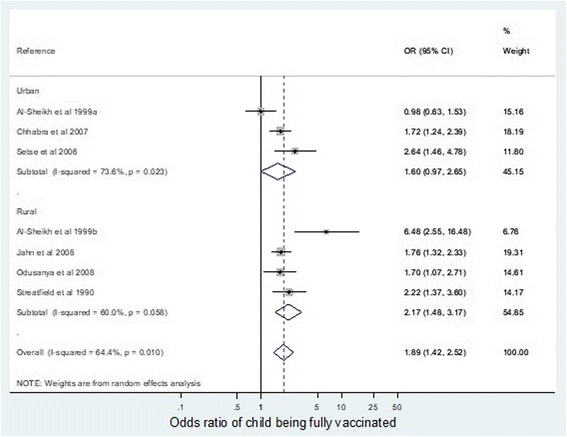
odds ratio of children being fully vaccinated if mother educated to a secondary level compared with no or primary education, according to setting

Within the setting subgroup analysis (Fig. 5), vaccination of children was most likely in highly educated women in rural areas, with an odds ratio 2.17 (95% CI 1.48–3.17). There was no statistically significant difference in the odds ratios between the rural and urban settings.

#### Timing

**Fig. 6:**
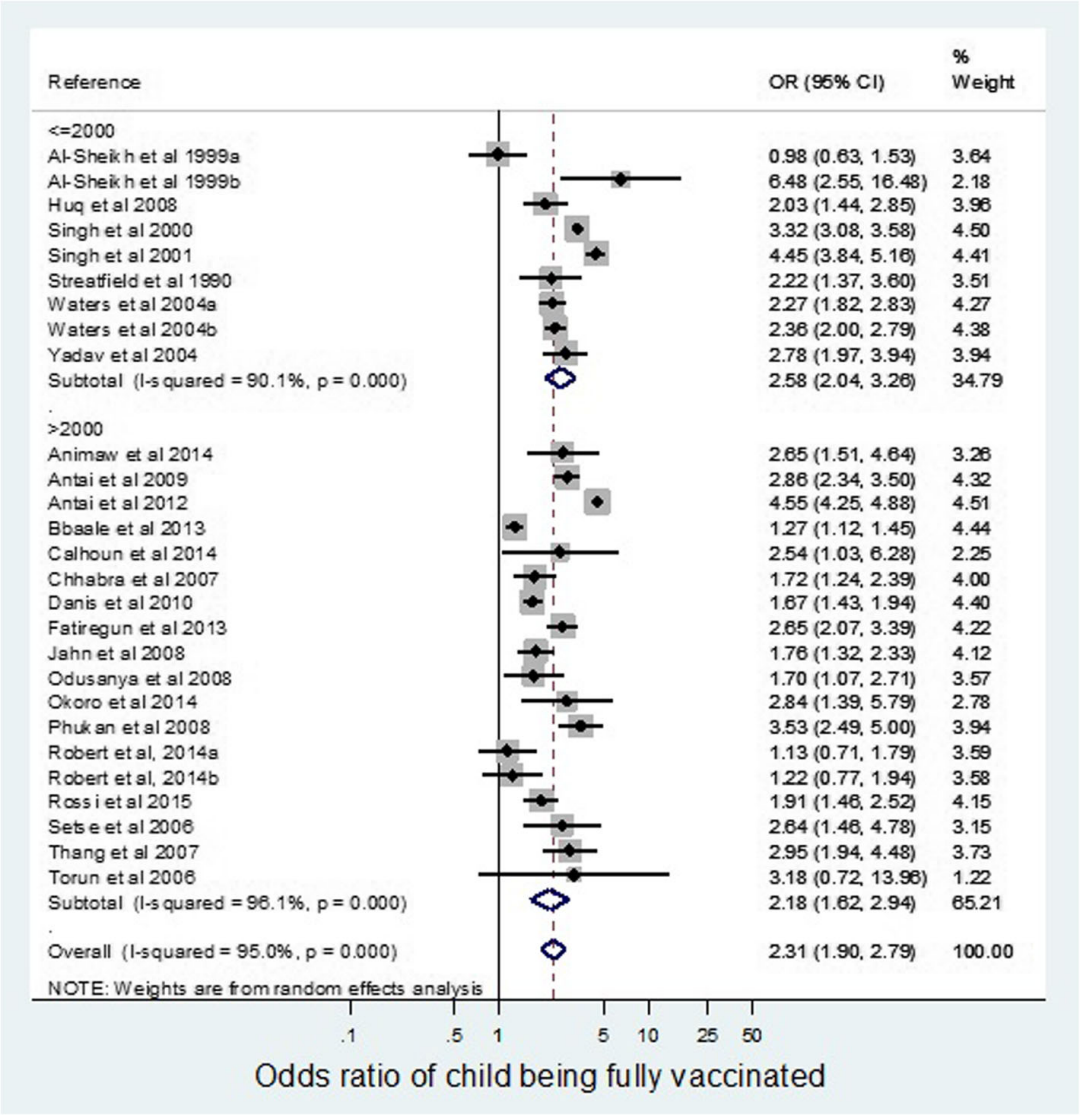
Odds ratio of children being fully vaccinated if mother educated to a secondary level compared with no or primary education, according to time period

As seen in Fig. 6, studies conducted before 2000 show an odds ratio of 2.58 (95% CI 2.04–3.26). The overall odds ratio for studies conducted from 2001 is 2.18 (95% CI 1.62–2.94). Although the odds of complete child vaccination are slightly lower in the later time period, there was no statistically significant difference in the odds ratios.

#### Summary estimate of vaccine completion by maternal education level

Collapsing of the different maternal education variables into the 4 categories, none, primary, secondary or tertiary education, to obtain the pooled estimate of the percentage of children fully vaccinated per strata is shown in Table 3. This demonstrates an increase in completion of vaccination as the maternal education level increases. Only 42.8% (95% CI 35.2–50.4) of children whose mothers had no education were fully vaccinated. This increases to 80.2% (95% CI 75.5–85.0) amongst children whose mothers had completed tertiary education. The pooled summary also shows that there is the overall prevalence of vaccination uptake was 57.8% (95% CI: 52.4–63.1).

**Table 3:** Pooled summary vaccination completion per education level

Maternal education level	Pooled child vaccination completion (%)	95% confidence interval	I-squared (%)
None	42.8	35.2–50.4	99.7
Primary	56.6	49.5–63.7	99.4
Secondary	64.3	56.1–72.5	99.2
Tertiary	80.2	75.5–85.0	89.3

However, there is significant heterogeneity between studies, as reflected in the I-squared values. This demonstrates that maternal education is not the only determinant of vaccination uptake.

## Discussion

### Summary

The primary finding of this review is that an increase in maternal education is correlated with increased childhood vaccination. However, the overall effect size of maternal education on vaccination completion cannot be concluded due to heterogeneity between the studies. Summary estimates of percentage of children fully vaccinated according to the level of maternal education showed a step-wise increase in overall percentages as maternal education increased from none to tertiary. Additionally, a significant difference was shown on the meta-analysis between literate and illiterate women, displaying that increased literacy has a beneficial impact on vaccination uptake.

This review also demonstrated a difference in the size of the effect seen between Asia and Africa compared to Europe. The higher odds ratio of maternal education on vaccination uptake in Asia and Africa may demonstrate that education plays a more important role in lower income countries. This could be due to societal development as areas with better education may also have improved healthcare access. Whilst the effect is lower in Europe, it is still positive. This demonstrates the importance of maternal education even in the presence of good health care programmes.

No difference in the effect of maternal education on vaccine uptake was found between urban and rural settings. It is of note that many of the studies were population based so are likely to be representative; however, two studies were conducted in a hospital setting so are less generalizable.

The results also show no difference in the effect of maternal education on vaccine uptake between time periods.

The heterogeneity seen between the results may be due to a number of other factors which may also affect vaccination uptake, such as availability of the immunizations, distance to healthcare facility, household income and maternal age which would confound the effect size [18]. Despite the presence of confounders, there remains a strong correlation between maternal education and child vaccination completion.

### Limitations

As with all studies, this review has some limitations. The main one was the exclusion of non-English papers which could potentially lead to language bias. Moreover, authors were not contacted for the raw data if the study had been excluded due to lack of published data in the required format.

In addition, condensing the maternal education variables may have hidden subtle patterns between the smaller jumps in education level. Furthermore, this meant that in studies with dichotomous variables of educated against not, and illiterate vs literate, the educated variable was also categorised as “none/primary” in the meta-analysis. Due to the differences in the settings of the studies, there was no universal standard for measuring level of education. In order to compare them in this review, they were categorised into set variables which contributed to the high heterogeneity.

### Implications of this review

This current review adds further evidence of the association between maternal education and child mortality reduction [19]. It is possible that child vaccination uptake is in fact one of the pathways for which this relationship is seen. It also shows that child vaccination uptake is not solely down to supply of vaccinations, and programs which aim to increase the dispersion of immunizations need to concentrate on these additional factors [20]. Furthermore, it adds to the current argument of the importance of educating women and gender equality [21]. Despite these associations this study does not answer the question of exactly how maternal education increases vaccine uptake. One link may be that increasing maternal education leads to more access to healthcare and therefore vaccine uptake. However, previous studies have theorised that maternal education, specifically literacy, enhance cognition and communication skills which encourage healthier lifestyle choices leading to lower childhood mortality [22].

The meta-analysis looking at literacy levels demonstrated that one of the potential mediators between maternal education and complete vaccination was maternal literacy. This is further supported by Balogun et al. who found that mothers who were literate, regardless of their education level, were more likely to vaccinate their children [23]. This therefore implies that improving the educational standards to ensure literacy will have a greater impact on increased childhood vaccination than simply increasing the throughput of girls in education.

Overall it is clear that female education is crucial in improving child health and should be considered when policies surrounding child health are implemented. Whilst this study cannot provide an overall total effect size of maternal education on child vaccination uptake, it does demonstrate that there is a consistently positive effect. This should be taken into consideration when global health policies aiming to increase the uptake of child vaccination are applied. It also highlights the importance of female education on wider factors other than self-improvement and the economy [19].

## Conclusions

This review highlights the positive effect of maternal education on childhood vaccination uptake across different continents, settings, and time periods.

It has been long established that childhood mortality is decreased by childhood vaccination [21]. This analysis identified that increased maternal education leads to increased childhood vaccination uptake and, in turn, will decrease childhood mortality.

## References

[1] World Health Organization (2009). State of the world ’ s vaccines and immunization.

[2] Vikram K, Vanneman R, Desai S (2012). Linkages between maternal education and childhood immunization in India. Soc Sci Med.

[3] Sullivan MC, Tegegn A, Tessema F, Galea S, Hadley C (2010). Minding the immunization gap: family characteristics associated with completion rates in rural Ethiopia. J Community Health.

[4] Antai D (2009). Inequitable childhood immunization uptake in Nigeria: a multilevel analysis of individual and contextual determinants. BMC Infect Dis.

[5] Sia D, Fournier P, Kobiané J-F, Sondo BK (2009). Rates of coverage and determinants of complete vaccination of children in rural areas of Burkina Faso (1998-2003). BMC Public Health.

[6] Shaikh S, Taj TM, Kazi A, Ahmed J, Fatmi Z (2010). Coverage and predictors of vaccination among children of 1-4 years of age in a rural sub-district of Sindh. J Coll Physicians Surg Pakistan.

[7] WHO, Immunization, Vaccines and Biologicals: Estimates of disease burden and cost-effectiveness. 2015. [Online]. Available: http://www.who.int/immunization/monitoring_surveillance/burden/estimates/en/. Accessed 22 Sept 2016.

[8] Rainey JJ, Watkins M, Ryman TK, Sandhu P, Bo A, Banerjee K (2011). Reasons related to non-vaccination and under-vaccination of children in low and middle income countries: findings from a systematic review of the published literature, 1999-2009. Vaccine.

[9] Effective public health practice project. Quality assessment tool for quantitative studies. Available from: http://www.ephpp.ca/PDF/Quality%20Assessment%20Tool_2010_2.pdf. Accessed 22 Sept 2016.

[10] Sanderson S, Tatt ID, Higgins JPT (2007). Tools for assessing quality and susceptibility to bias in observational studies in epidemiology: a systematic review and annotated bibliography. Int J Epidemiol.

[11] Thomas BH, Ciliska D, Dobbins M, Micucci S (2004). A process for systematically reviewing the literature: providing the research evidence for public health nursing interventions. Worldviews Evid Based Nurs.

[12] Greenhalgh T (1997). How to read a paper: papers that summarise other papers (systematic reviews and meta-analyses). BMJ.

[13] Adekola O. Language, literacy and learning in primary schools. Washington: World Bank; 2007. p. 5-16.

[14] Dersimonian R, Laird N (1986). Meta-analysis in clinical trials. Stat Med.

[15] StataCorp (2013). Stata statistical software: release 13.

[16] Abuya BA, Onsomu EO, Kimani JK, Moore D (2011). Influence of maternal education on child immunization and stunting in Kenya. Matern Child Health J.

[17] Al-Sheikh OG, Al-Samarrai JI, Al-Sumaidaie MM, Mohammad SA, Al-Dujaily AA (1999). Immunization coverage among children born between 1989 and 1994 in Saladdin governorate, Iraq. East Mediterr Heal J.

[18] Danis K, Georgakopoulou T, Stavrou T, Laggas D, Panagiotopoulos T (2010). Socioeconomic factors play a more important role in childhood vaccination coverage than parental perceptions: a cross-sectional study in Greece. Vaccine.

[19] Gakidou E, Cowling K, Lozano R, Murray CJ (2010). Increased educational attainment and its effect on child mortality in 175 countries between 1970 and 2009: a systematic analysis. Lancet.

[20] Antai D. Gender inequities, relationship power, and childhood immunization uptake in Nigeria: A population-based cross-sectional study. Int J Infect Dis. 2012;16(2):136-45. 10.1016/j.ijid.2011.11.004 22197748

[21] United Nations, The Millennium Development Goals Report. 2012. [Online]. Available: http://www.un.org/millenniumgoals/pdf/MDG Report 2012.pdf. Accessed 22 Sept 2016.

[22] Smith-Greenaway E (2013). Maternal reading skills and child mortality in Nigeria: a reassessment of why education matters. Demography.

[23] Balogun SA, Yusuff HA, Yusuf KQ, Al-Shengiti AM, Balogun MT, Tettey P (2017). Maternal education and child immunization: the mediating roles of maternal literacy and socioeconomic status. Pan Afr Med J.

[24] Animaw W, Taye W, Merdekios B, Tilahun M, Ayele G. Expanded program of immunization coverage and associated factors among children age 12–23 months in Arba Minch town and Zuria District, Southern Ethiopia, 2013. BMC Public Health. 2014;14(1):464. 10.1186/1471-2458-14-464 PMC4032449 24884641

[25] Bbaale E. Factors influencing childhood immunization in Uganda. J Health Popul Nutr. 2013;31(1):118-29. 10.3329/jhpn.v31i1.14756 PMC3702366 23617212

[26] Branco F, Pereira T, Delfino B, Braña A, Oliart-Guzmán H, Mantovani S, Martins A, de Menezes Oliveira C, Ramalho A, Codeço C, da Silva-Nunes M. Socioeconomic inequalities are still a barrier to full child vaccine coverage in the Brazilian Amazon: a cross-sectional study in Assis Brasil, Acre, Brazil. Int J Equity Health. 2014;13(1):118. 10.1186/s12939-014-0118-y PMC4256802 25428334

[27] Brenner R, Simons-Morton B, Bhaskar B, Das A, Clemens J (2001). Prevalence and predictors of immunization among Inner-City infants: a birth cohort study. Pediatrics.

[28] Calhoun L, van Eijk A, Lindblade K, Odhiambo F, Wilson M, Winterbauer E, Slutsker L, Hamel M (2013). Determinants and coverage of vaccination in children in western Kenya from a 2003 cross-sectional survey. Am J Trop Med Hyg.

[29] Chhabra P, Nair P, Gupta A, Sandhir M, Kannan A (2007). Immunization in urbanized villages of Delhi. Indian J Pediatr.

[30] Elliott C, Farmer K (2006). Immunization status of children under 7 years in the Vikas Nagar area, North India. Child Care Health Dev.

[31] Fatiregun A, Okoro A (2012). Maternal determinants of complete child immunization among children aged 12–23 months in a southern district of Nigeria. Vaccine.

[32] Fatiregun A, Adebowale A, Ayoka R, Fagbamigbe A (2013). Assessing full immunisation coverage using lot quality assurance sampling in urban and rural districts of southwest Nigeria. Trans R Soc Trop Med Hyg.

[33] Huq MN, Tasnim T (2008). Maternal education and child healthcare in Bangladesh. Matern Child Health J.

[34] Jahn A, Floyd S, Mwinuka V, Mwafilaso J, Mwagomba D, Mkisi R, Katsulukuta A, Khunga A, Crampin A, Branson K, McGrath N, Fine P (2008). Ascertainment of childhood vaccination histories in northern Malawi. Tropical Med Int Health.

[35] Kidane T, Tekie M. Factors influencing child immunization coverage in a rural District of Ethiopia, 2000. Ethiop J Health Dev. 2003;17(2):105-10.

[36] Koumaré A, Traore D, Haidara F, Sissoko F, Traoré I, Dramé S, Sangaré K, Diakité K, Coulibaly B, Togola B, Maïga A (2009). Evaluation of immunization coverage within the expanded program on immunization in Kita circle, Mali: a cross-sectional survey. BMC Int Health Hum Rights.

[37] Kumar D, Aggarwal A, Gomber S (2010). Immunization status of children admitted to a tertiary-care hospital of north India: reasons for partial immunization or non-immunization. J Health Popul Nutr.

[38] Luman ET, McCauley MM, Shefer A, Chu SY (2003). Maternal characteristics associated with vaccination of young children. Pediatrics.

[39] Mohamud A, Feleke A, Worku W, Kifle M, Sharma H. Immunization coverage of 12–23 months old children and associated factors in Jigjiga District, Somali National Regional State, Ethiopia.BMC Public Health. 2014;14(1):865. 10.1186/1471-2458-14-865 PMC4158082 25146502

[40] Odusanya O, Alufohai E, Meurice F, Ahonkhai V. Determinants of vaccination coverage in rural Nigeria. BMC Public Health. 2008;8(1):381. 10.1186/1471-2458-8-381 PMC2587468 18986544

[41] Okoro J, Ojinnaka N, Ikefuna A, Onyenwe N (2015). Sociodemographic influences on immunization of children with chronic neurological disorders in Enugu, Nigeria. Trials Vaccinology.

[42] Pati S, Feemster K, Mohamad Z, Fiks A, Grundmeier R, Cnaan A (2010). Maternal health literacy and late initiation of immunizations among an Inner-City birth cohort. Matern Child Health J.

[43] Phukan R, Barman M, Mahanta J (2008). Factors associated with immunization coverage of children in Assam, India: over the first year of life. J Trop Pediatr.

[44] Robert E, Dramaix M, Swennen B (2014). Vaccination coverage for infants: cross-sectional studies in two regions of Belgium. Biomed Res Int.

[45] Rossi R (2015). Do maternal living arrangements influence the vaccination status of children age 12–23 months? A data analysis of demographic health surveys 2010–11 from Zimbabwe. PLoS One.

[46] Schoeps A, Ouédraogo N, Kagoné M, Sié A, Müller O, Becher H (2013). Socio-demographic determinants of timely adherence to BCG, Penta3, measles, and complete vaccination schedule in Burkina Faso. Vaccine.

[47] Setse R (2006). HIV-1 infection as a risk factor for incomplete childhood immunization in Zambia. J Trop Pediatr.

[48] Singh P, Yadav RJ (2000). Immunization status of children of India. Indian Paediatr.

[49] Singh P, Yadav R (2001). Immunisation status of children in BIMARU states. Indian J Pediatr.

[50] Som S, Pal M, Chakrabarty S, Bharati P (2010). Socioeconomic impact on child immunisation in the districts of West Bengal, India. Singap Med J.

[51] Streatfield K, Singarimbun M, Diamond I (1990). Maternal education and child immunization. Demography.

[52] Thang NM, Bhushan I, Bloom E, Bonu S (2006). Child immunization in Vietnam: situation and barriers to coverage. J Biosoc Sci.

[53] Torun S, Bakırcı N. Vaccination coverage and reasons for non-vaccination in a district of Istanbul. BMC Public Health. 2006;6(1):125. 10.1186/1471-2458-6-125 PMC1464125 16677375

[54] Waters HR, Dougherty L, Tegang SP, Tran N, Wiysonge CS, Long K (2004). Coverage and costs of childhood immunizations in Cameroon. Bull World Health Organ.

[55] Yadav R, Singh P (2004). Immunisation status of children and mothers in the state of Madhya Pradesh. Hindu.

